# Value of transbronchial needle aspiration combined with a rapid on-site evaluation of cytology in the diagnosis of pulmonary lesions

**DOI:** 10.3389/fmed.2022.922239

**Published:** 2022-10-05

**Authors:** Long Liang, Hongxia Duan, Huiyuan Zhu, Huiqiong Yang, Xuan Li, Changhui Wang, Shuanshuan Xie

**Affiliations:** ^1^Department of Respiratory Medicine, Shanghai Tenth People’s Hospital, Tongji University School of Medicine, Shanghai, China; ^2^Department of Oncology, Affiliated Hospital of Nantong University, Nantong, China; ^3^Department of Pathology, Shanghai Tenth People’s Hospital, Tongji University School of Medicine, Shanghai, China

**Keywords:** rapid on-site evaluation, endobronchial ultrasound-guided transbronchial needle aspiration, pulmonary lesions, diagnostic yield, complications

## Abstract

**Background:**

The diagnostic value of rapid on-site evaluation (ROSE) of cytology during endobronchial ultrasound-guided transbronchial needle aspiration (EBUS-TBNA) remains controversial. The purpose of this study was to validate the value of ROSE during the EUBS-TBNA procedure in the diagnosis of pulmonary lesions (PLs).

**Methods:**

Enrolled in this study were 260 patients with nodules, masses, cavities, or inflammatory lesions on pulmonary CT images. They were assigned to undergo EBUS-TBNA with ROSE (*n* = 134) or without ROSE (*n* = 126). The diagnostic results of ROSE during EBUS-TBNA and the final pathologic reports were analyzed and compared by utilizing SPSS21.0 software to evaluate the sensitivity, specificity, positive predictive value (PPV), and negative predictive value (NPV). In addition, we further explored whether the ROSE method during EBUS-TBNA would improve the diagnostic yield and reduce the incidence of complications.

**Results:**

The overall diagnostic yield of EBUS-TBNA for malignant diseases in the ROSE and the non-ROSE group were 29.9 and 11.1%, respectively. The sensitivity, specificity, PPV and NPV of the ROSE method during EBUS-TBNA were 97.4, 96.9, 92.5, and 98.90%, respectively. The result of the chi-square test effectively proved that ROSE operation during EBUS-TBNA contributes to the diagnosis of malignancy compared with the non-ROSE group (χ^2^ = 13.858, *P* < 0.001). The number of punctures in the ROSE group was significantly lower than that in the non-ROSE group (*P* < 0.001).

**Conclusion:**

ROSE examination during EBUS-TBNA could effectively improve the diagnostic yield of malignant diseases compared with the non-ROSE group and reduce the number of intraoperative punctures, which is a clinical application worth popularizing.

## Introduction

Pulmonary nodules are one of the common radiological manifestations of pulmonary lesions (PLs). In recent years, the incidence of lung cancer has increased significantly worldwide ranking first among malignant diseases and has become a major disease endangering people’s life and health (1). Interventional bronchoscopy is one of the most extensively used clinical approaches for the diagnosis of early lung cancer and other respiratory system-related diseases (2). Transbronchial needle aspiration (TBNA) has been widely used for the determination of focal properties adjacent to the trachea and bronchial walls, diagnosis of pulmonary hilar, and mediastinal lymph nodes, and staging of lung cancer (3). Unfortunately, tissue lesions could not be visualized in a real-time manner during the conventional TBNA procedure, thus increasing the possibility of causing accidental injury to the normal organs around the airway. With the advent of ultrasound probes mounted at the front end of the microscope, it has been made possible to enter the airway for ultrasound scanning, thus enabling the integration of the respective advantages of the above two techniques, known as endobronchial ultrasound-guided transbronchial needle aspiration (EBUS-TBNA) (4, 5).

Pathological examination remains the gold standard for the diagnosis of pulmonary malignant tumors. However, difficulty in sampling and time-consuming of the diagnostic procedure may delay the treatment, extend the length of hospitalization stay and increase the medical cost. In contrast, rapid on-site evaluation (ROSE) of cytology during EBUS-TBNA can realize the rapid diagnosis of lesions obtained and avoid the time-consuming defects in the pathological examination.

Several studies have reported the joint use of ROSE based on EBUS-TBNA (6), saying that the combined use of the above two technologies could provide the examiner with useful information on whether the puncture is successful, to enable him/her to evaluate the quality of specimens in a real-time manner, and to decide whether a repeated operation is necessary or not (7–9). However, there is still no agreement on whether ROSE technology could improve the diagnostic yield of lung malignancies. This retrospective study aimed to evaluate whether the ROSE method during EBUS-TBNA could effectively improve the diagnostic yield of lung malignancies and reduce surgery-related complications.

## Patients and methods

### Screening of patients

**FIGURE 1:**
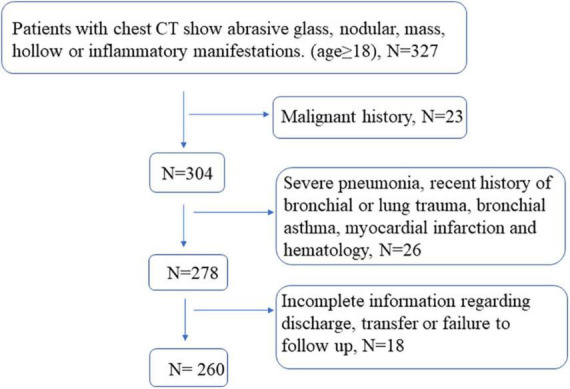
Standard flowchart for patient screening.

This research was approved by the ethics committee of Shanghai Tenth People’s Hospital affiliated with Tongji University (Shanghai, China; No. SHSY-IEC-4.1/20–21/01). All methods were carried out under the relevant guidelines and regulations. The study enrolled 260 patients who had undergone EBUS-TBNA with ROSE (*n* = 134) or without ROSE (*n* = 126) in the bronchoscopy room of the said hospital between 2018 and 2020. The clinical data of the patients in the two groups including sex, age, smoking history, accompanying symptoms, underlying diseases, and the length of hospitalization stay are presented in Table 1. Contraindications to bronchoscopy were examined before operation in all patients. In addition, routine examinations including electrocardiography, coagulation time, electrolytes, and infectious diseases were also performed before the operation. The inclusion criteria were patients older than 18 years whose chest CT scan showed ground glass or ordinary nodules, masses, and cavity or inflammatory manifestations. Not suitable for this procedure were patients with major diseases such as cachectic, a recent history of bronchial or lung trauma, bronchial asthma, myocardial infarction, and hematological diseases. Patients with incomplete information regarding discharge, transfer, or failure to follow up were also excluded (Figure 1).

**TABLE 1:** Baseline characteristics of patients.

	ROSE (*n* = 134)	Non-ROSE (*n* = 126)	χ^2^	*P*-value
Age (years, mean ± SD)	60.75 ± 12.75	60.56 12.75	/	0.904
Sex (*n*, %)			0.028	0.900
Male	79 (59.0%)	73 (57.9%)		
Female	55 (41.0%)	53 (42.1%)		
Smoking history (*n*, %)			0.334	0.575
No	101 (75.4%)	91 (72.2%)		
Yes	33 (24.6%)	35 (27.8%)		
**Symptoms**				
Dyspnea (*n*, %)			1.572	0.249
No	121 (90.3%)	119 (94.4%)		
Yes	13 (9.7%)	7 (5.6%)		
Cough (*n*, %)			0.800	0.442
No	80 (59.7%)	82 (65.1%)		
Yes	54 (40.3%)	44 (34.9%)		
Fever (*n*, %)			0.098	0.862
No	113 (84.3%)	108 (85.7%)		
Yes	21 (15.7%)	18 (14.3)		
Joint pain (*n*, %)			2.363	0.158
No	130 (97.0%)	117 (92.9%)		
Yes	4 (3.0%)	9 (7.1%)		
**Basic diseases**				
Hypertension (*n*, %)			1.335	0.303
No	107 (79.9%)	93 (73.8%)		
Yes	27 (20.1%)	33 (26.2)		
Diabetes (*n*, %)			1.096	0.327
No	114 (85.1%)	101 (80.2%)		
Yes	20 (14.9%)	25 (19.8%)		
Hypertension+ diabetes (*n*, %)			0.01	1.000
No	124 (92.5%)	117 (92.9%)		
Yes	10 (7.5%)	9 (7.1%)		
The length of hospitalization stay (mean ± SD)	9.03 ± 4.16	8.12 ± 3.55	/	0.059

ROSE, Rapid On-Site Evaluation.

### Preoperative preparations and postoperative management

The patient was kept nil for at least 6 h before the procedure. Patients were placed in a supine position; intravenous access was established. Midazolam was administered by a pulmonologist for conscious sedation. Oxygen was given to the patient through the nasal catheter, and the basic life indicators including blood pressure, pulse, respiratory frequency, and oxygen saturation were monitored routinely during the whole operation process. A chest X-ray and routine blood examination were performed if uncomfortable symptoms or abnormal signs developed after this operation. For malignancies that failed to be detected by EBUS-TBNA, further CT-guided transthoracic puncture or open-chest surgery was required to obtain a reliable pathological tissue diagnosis.

### A summary of the sample processing

**FIGURE 2:**
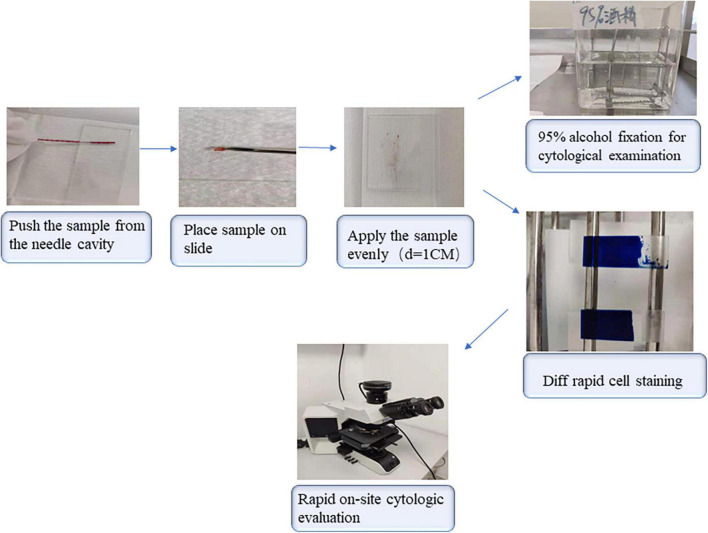
A flow diagram of procedure details.

The specimen absorbed into the needle cavity was gently pushed by the central tube core needle and spread over the slide as evenly as possible in a circle about 1 cm in diameter. A portion of the visible tissue fragment collected on the glass slide was transferred into a single container containing formalin for later histological examination, making sure that the tissue was immersed completely in formalin; the basic information of the specimen was marked on the surface of the bottle. The remaining specimen was divided into two sections and smeared onto two slides. The specimen remaining in the needle cavity and catheter cavity was washed down with normal saline and collected for microbiological analysis. The specimens in ROSE and non-ROSE groups were treated differently. One of the slides in the ROSE group was stained with Diff-quick cell staining solution AB (Zhuhai Beso Biotechnology Co., Ltd., Zhuhai, China) to observe the cell morphology and composition under the microscope by two experienced pathologists, who then decided whether sampling was completed successfully, or should be re-performed elsewhere. The other slide was placed in a bottle containing 95% alcohol for subsequent Papanicolaou staining and cytological examination. If abnormal cells were detected by ROSE, 2–3 more tissues needed to be taken at the same site. Otherwise, sampling would be continued in other sites to find whether there were diagnostic specimens that we needed. All non-ROSE specimens were fixed with 95% alcohol for cytological examination. The above processes are shown in Figure 2.

### Diagnosis

The histological and cytological specimens were interpreted by two experienced pathologists. The final diagnostic results were determined by the pathological results of the histological biopsy. For suspicious cases or non-specific diagnostic results, thoracoscopic examination, mediastinal examination, or other surgical procedures were carried out according to the patient’s wishes. For patients who were temporarily unwilling to undergo invasive examinations, preliminary conclusions could be obtained after considering the clinical symptoms, imaging findings, and laboratory examinations. A follow-up examination was performed once at least 6 months after EBUS-TBNA to further verify the original judgment according to the therapeutic effect. The diagnostic yield, sensitivity, specificity, positive predictive value (PPV), negative predictive value (NPV), and Youden index were calculated according to ROSE and pathological results.

### Statistical analysis

The SPSS21.0 software was used for statistical analysis. Continuous variables were analyzed using a *t*-test, and dichotomous variables were analyzed using Pearson’s χ^2^-test or Fisher’s exact test. Results were considered statistically significant only when the *P*-value was less than 0.05.

## Results

### Baseline characteristics of the patients

Our statistics showed no significant difference in sex (*P* = 0.900) and age (*P* = 0.904) between the two groups, and the number of smokers was approximately the same (33 vs. 35, *P* = 0.575). The main symptoms were cough (54 vs. 44), fever (21 vs. 18), dyspnea (13 vs. 7), and combined chest pain (4 vs. 9) in ROSE and non-ROSE groups. And there was no significant difference in these symptoms between the two groups (χ^2^ = 0.800, *P* = 0.442; χ^2^ = 0.098, *P* = 0.862; χ^2^ = 1.572, *P* = 0.249; χ^2^ = 2.363, *P* = 0.158, respectively). The most common underlying diseases in both groups were hypertension and diabetes. No significant statistical difference in terms of underlying diseases between the two groups (χ^2^ = 1.335, *P* = 0.303; χ^2^ = 1.096, *P* = 0.327; respectively). The average length of hospitalization stay was 9.03 days in the ROSE group and 8.12 days in the non-ROSE group, showing no significant difference between the two groups (*P* = 0.059) (as shown in Table 1). Data showed no significant statistical difference in the baseline characteristics including sex, age, smoking history, symptoms, associated underlying diseases, and hospitalization days between ROSE and non-ROSE groups.

### Size, nature, and distribution characteristics of the lesions

We further analyzed the size, nature, and distribution location of lesions in the lung lobe between ROSE and non-ROSE groups. The specific distribution of lesions involved the right upper, right middle, right lower, left upper, left lingual, left lower, left pulmonary valve, lower or upper lobe of both lungs and all lungs. There were no significant differences in size (*P* = 0.669) and distribution (*P* = 0.139) of the lesions between the two groups. The lesions were divided into five categories according to their nature and characteristics: grinding nodules, common nodules, bumps, cavities and inflammatory exudations, which were significantly different between the two groups (*P* = 0.008) (Table 2).

**TABLE 2:** Size, nature and distribution characteristics of the lesions.

	ROSE	Non-ROSE	χ^2^	*P*-value
Lesion size (cm, mean ± SD)	1.51 ± 2.15	1.40 ± 1.82	/	0.669
Nature of lesion			13.650	0.008*
Grinding lesion	18	31		
Nodules	42	47		
Bump	20	19		
Cavity	5	0		
Inflammation	49	29		
Location of lesion			13.563	0.139
Right upper lobe	27	36		
Right middle lobe	12	12		
Right lower lobe	26	19		
Left upper division	20	27		
Left lingual lobe	7	4		
Left lower lobe	14	18		
Left pulmonary valve	1	1		
Lower lobe of both Lungs	4	1		
Upper lobe of both lungs	6	2		
Both lungs	17	6		

ROSE, Rapid On-Site Evaluation.

*P-value less than 0.05 is statistically significant difference.

### Preliminary diagnosis and classification of benign and malignant diseases by using the method of rapid on-site evaluation during endobronchial ultrasound-guided transbronchial needle aspiration

Forty (29.9%) malignant nodules were detected in the ROSE group, including 26 cases of adenocarcinoma (AD), 12 cases of squamous cell carcinoma (SCC), and 2 cases of small cell lung cancer (SCLC). In addition, 2 cases of hamartoma, 1 case of synovial sarcoma, 1 case of tuberculosis, 3 cases of pulmonary abscesses, and 5 cases of fungal infections were detected as benign lesions. In the non-ROSE group, only 14 patients (11.1%) were diagnosed as malignant nodules by pathology, including 5 cases of AD, 4 cases of SCC, 4 cases of SCLC, and 1 case of cancer *in situ*. Furthermore, 1 case of dysplasia, 3 cases of lung abscess, and 3 cases of fungal infections were diagnosed as benign lesions in this group. Inflammatory nodules occupied a very large proportion in both the ROSE and non-ROSE groups (82 vs. 105) (as shown in Supplementary Figure 1). The statistical method of the chi-square test was adopted in our study, demonstrating that the application of ROSE technology during EBUS-TBNA contributes to the identification of benign and malignant tumors compared with the non-ROSE group as described in Table 3 (χ^2^ = 13.858, *P* < 0.001).

**TABLE 3:** Preliminary diagnosis and classification of benign and malignant diseases by using the method of ROSE during EBUS-TBNA.

Characteristics	ROSE (*n* = 134)	Non-ROSE (*n* = 126)	χ^2^	*P*
Malignant(*n*, %)	40 (29.9%)	14 (11.1%)	13.858	<0.001***
Adenocarcinoma	26	5		
Squamous-cell carcinoma	12	4		
Small cell lung cancer	2	4		
Tis	0	1		
Benign (*n*,%)	94 (70.1%)	112 (88.9%)		
Hamartoma	2	0		
Dysplasia	0	1		
Synovial sarcoma	1	0		
Tuberculosis	1	0		
Abscess	3	3		
Mycotic infection	5	3		
Non-representative samples	82	105		

ROSE, Rapid On-Site Evaluation.

***P-value less than 0.001 is statistically significant difference.

### Accuracy of diagnosis

**FIGURE 3:**
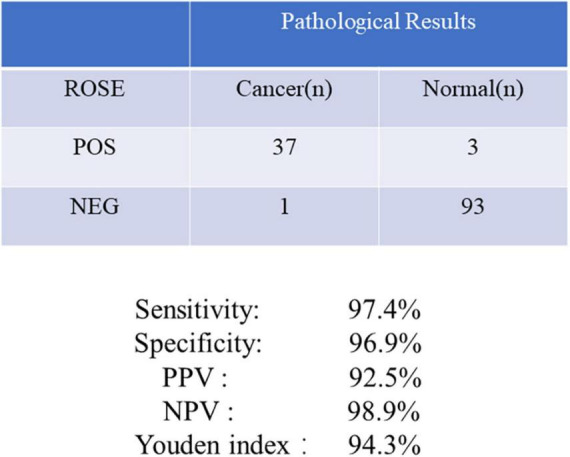
Comparison of the ROSE diagnosis during EBUS-TBNA with the final pathological findings.

The overall diagnostic yield of EBUS-TBNA for malignant diseases in the ROSE and non-ROSE groups were 29.9 and 11.1%, respectively (Table 3). It fully demonstrates that ROSE technology during EBUS-TBNA has an advantage in improving the diagnostic yield of malignant diseases compared to the non-ROSE group. The diagnostic accuracy of ROSE in PLs relative to the pathological results was calculated. The results showed that the sensitivity, specificity, PPV, and NPV of ROSE during EBUS-TBNA were 97.4, 96.9, 92.5, and 98.9%, respectively, and the Youden index was 94.3%, showing a good agreement with the pathological diagnosis (Figure 3).

### Complications

The number of punctures for major target lesions in the ROSE group was significantly lower than that in the non-ROSE group (*P* < 0.001). No pneumothorax occurred in the ROSE group vs. 2 cases in the non-ROSE group. Hemoptysis occurred in 86 cases in the ROSE group vs. 84 cases in the non-ROSE group, accounting for about 75% of the number of cases in each group and showing no statistically significant difference between the two groups (Table 4). None of the patients required further intensive care treatment.

**TABLE 4:** Comparison of complications between ROSE and non-ROSE groups.

Complications	ROSE (*n*)	Non-ROSE (*n*)	*P*-value
Pneumothorax	No	2	/
Hemoptysis	86	84	0.402
Number of punctures (mean ± SD)	2.07 ± 0.26	3.23 ± 0.42	<0.001***

ROSE, Rapid On-Site Evaluation.

***P-value less than 0.001 is statistically significant difference.

### Cytomorphological characteristics of the different lung cancer subtypes

**FIGURE 4:**
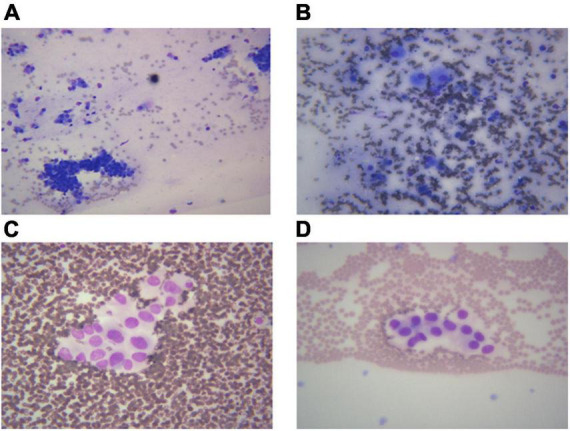
Images from different cancer types. According to the morphological characteristics of cells, ROSE can achieve accurate classification of lung cancer subtypes. **(A)** Squamous cell carcinoma, 400×; **(B)** adenocarcinoma derived from gastrointestinal cancer metastasis, 400×; **(C,D)** adenocarcinoma, 400×.

**FIGURE 5:**
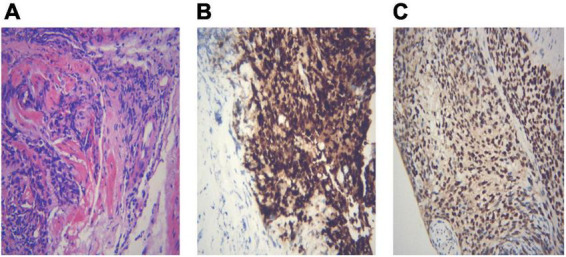
An example of histological biopsy from a poorly differentiated squamous cell carcinoma (SCC). **(A)** Shows typical poorly differentiated morphological features of SCC. The tumor cells are large and flat, with fusiform deeply stained nuclei and abundant cytoplasm (HE 400×). The diagnosis is confirmed by the immunoreactivity for P40 (**B**, IHC 400×) and P63 expression (**C**, IHC 400×). **(A)** Poorly differentiated SCC (HE 400×); **(B)** P40 400×; **(C)** P63 400×.

**FIGURE 6:**
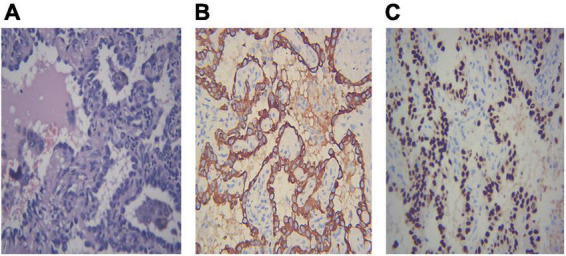
Demonstration of histological biopsy from adenocarcinoma. Under the microscope, adenocarcinoma consists of cubic and columnar cells with large or irregular nuclear nuclei and distinct nucleoli (**A**, HE 400×). Tumor cells show positive immunoreactivity for cytokeratin 7 and TTF-1 (**B,C** IHC 400×). **(A)** Adenocarcinoma HE 400×; **(B)** CK7 400×; **(C)** TTF-1 400×.

**FIGURE 7:**
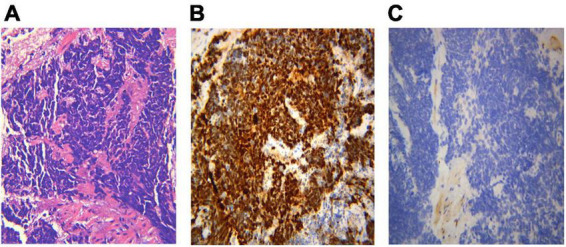
Histological biopsy of a case of small cell lung cancer (SCLC). HE staining presents the morphological features: tumor cells are small in size and round or oval in shape, mimicking lymphocytes, with rare cytoplasm, deeply stained nuclei and invisible nucleoli (**A**, HE 400×). Abnormal cells are positive for TTF-1 immunoreactivity (**B**, IHC 400×), and negative for CD56 (**C**, IHC 400×). **(A)** Small cell lung cancer HE 400×; **(B)** TTF-1 400×; **(C)** CD56(-) 400×.

**FIGURE 8:**
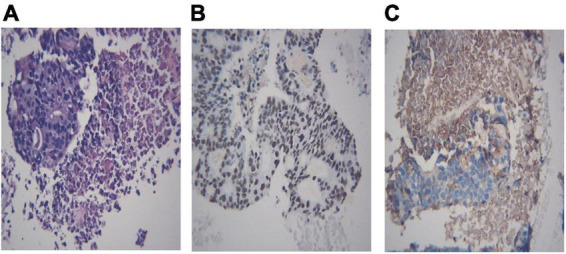
Histological biopsy of a pulmonary adenocarcinoma with metastasis from gastrointestinal neoplasm. Tumor cells are grouped in clusters (**A**, HE 400×) and positive for Brg-1 (**B**, IHC 400×) and Claudin-4 (**C**, IHC 400×). **(A)** Metastasis from gastrointestinal neoplasm, HE 400×; **(B)** Brg-1 400×; **(C)** Claudin-4 400×.

Different lung cancers show their unique cytological features under the microscope. ROSE technique could achieve accurate subtyping of lung cancer based on the cytomorphologic features, especially in well-differentiated tumors. The general microscopic cytology morphology of SCC and AD are shown in Figure 4. The characteristics of the tumor cells are specifically depicted as follows: SCC cells adhered closely and showed clusters, with abnormal morphology of cancer cells and nuclei. AD cells gathered into closely distributed small cell clumps, with clear nucleoli and abundant cytoplasm. Figure 5 presents typical poorly differentiated morphological features of SCC by HE staining. The tumor cells were large and flat, with fusiform deeply stained nuclei and abundant cytoplasm. The diagnosis was confirmed by the immunoreactivity for P40 and P63 expression. Microscopically, HE staining under the microscope demonstrated that AD cells were mainly composed of cubic and columnar cells with large or irregular nuclear nuclei. Tumor tissues showed positive cytokeratin 7 and TTF-1 immunoreactivity (Figure 6). HE staining presented the following morphological features of SCLC tumor cells: small in size and round or oval in shape, mimicking lymphocytes, with rare cytoplasm and deeply stained nuclei. Abnormal cells were positive for TTF-1 immunoreactivity and negative for CD56 (Figure 7). Histological biopsy of pulmonary AD derived from metastatic gastrointestinal neoplasms is shown in Figure 8. Tumor cells were distributed in clusters and positive for Brg-1 and Claudin-4. The impossibility to afford rapid evaluation and analysis is the main disadvantage of using histological materials for pathological diagnosis. Conversely, the advantages are more attractive in that it can provide more tissue materials for immunohistochemical analysis, thus ensuring more convincing diagnostic results of certain tumor types.

## Discussion

We collected multiple sample types in this study, including grinding glass lesions, nodules, masses, cavities, and inflammatory lesions. Our data suggest that there was a trend toward a higher diagnostic yield for malignant diseases in the ROSE group compared to the non-ROSE group. And we concluded that the diagnostic yield of the ROSE method during the EBUS-TBNA procedure for PLs was close to the result of pathological diagnosis. There is a good agreement between the above two methods. Another conclusion with clinical guidance that was also drawn from our study is that the number of punctures in the ROSE group was significantly lower than that in the non-ROSE group. There was no significant difference in hemoptysis between the two groups. The report of our study effectively demonstrated that the ROSE during EBUS-TBNA was a valuable diagnostic method to determine the nature of suspicious lesions.

Our findings have also been validated in several reports abroad. Some scholars have demonstrated a high rate of agreement between ROSE combined with EBUS-TBNA and the final pathological result and proved the effectiveness of ROSE in determining the quality and quantity of specimens (10, 11). Davenport (12) reported that ROSE could increase the detection rate of specimens containing malignant cells by comparing 73 aspirates using the technique with 134 routinely processed aspirates. Diette et al. (13) demonstrated that the ROSE technique could significantly improve the diagnostic yield in the evaluation of lung nodules or masses and/or hilar or mediastinal lymphadenopathies. The application value of ROSE in another interventional diagnosis for pulmonary malignant diseases should also be appreciated. A randomized trial performed by Mondoni et al. (14) showed that the ROSE method increased the cytological diagnostic sensitivity of TBLB from 76 to 97%. Lin et al. (15) collected information from 336 patients undergoing EBUS-TBB surgery, and the application of the ROSE method significantly improved diagnostic accuracy compared with EBUS-TBB without ROSE (88.4% vs. 68.0%, *P* < 0.001). Another study (16) showed a better diagnostic yield from 89.2% without ROSE to 92.1% with ROSE in sampling hilar–mediastinal lymphadenopathies in lung cancer. The utility of ROSE during EBUS-TBNA for lymph node staging and the mode of surgical resection in lung cancer has also been demonstrated in relevant studies (17, 18). This is mainly due to the stable and rapid diagnostic pattern of ROSE and the small demand for sample size. The occurrence of early cancer is often accompanied by the phenomenon of adjacent lymphadenopathy (19), and the clinical value of ROSE is to take full advantage of less sampling and fast diagnosis to achieve rapid identification of abnormal cells in lymph nodes (13, 20–24). ROSE enables rapid and accurate interpretation of cancerous tissues and surrounding cancerous tissues and then instructs clinicians to adjust the direction of puncture, which can reduce intraoperative traumas and facilitate the detection of diseased tissues (25–27), contributing to the choice of surgical modes (28–31). Although the clinical utility of ROSE has been reported in kinds of literature, it is still a controversial question on whether can be popularized in clinical practice. Some argue that ROSE does not benefit patients by improving the diagnostic yield for malignant diseases and is limited in clinical application due to the lack of professional pathologists (32, 33). However, our study strongly demonstrated that the ROSE method could improve the diagnostic yield of lung neoplastic diseases and not increase the risk of complications during the operation; instead, it significantly reduced the frequency of punctures, eliminate unnecessary surgical traumas and bring more benefits to patients compared with the non-ROSE group. The good agreement between the ROSE diagnosis and the final pathological results is also well demonstrated, laying a solid theoretical foundation for the clinical application of ROSE. In the current era of precision medicine, ROSE services have become an important part of achieving rapid diagnosis and decision-making.

There are still some shortcomings in our study. First, the study was conducted in a single-center, which may affect the universality of the conclusion. In addition, this is a retrospective study, which may bring about bias in data collection and reliability of the results. Finally, the number of cases summarized in this study was relatively small.

## Conclusion

In conclusion, the prevalence of the ROSE method during EBUS-TBNA is expected to upgrade the level of interventional bronchoscopy to a new profile, and to further meet the patients’ needs for high-quality and accurately targeted medical services, and promote the development of medical undertakings around the world.

## Data availability statement

The raw data supporting the conclusions of this article will be made available by the authors, without undue reservation.

## Ethics statement

The studies involving human participants were reviewed and approved by the Ethics Committee of Shanghai Tenth People’s Hospital affiliated with Tongji University. The patients/participants provided their written informed consent to participate in this study.

## Author contributions

LL, HD, HZ, HY, XL, CW, and SX: conception and design and drafting of the manuscript. All authors: acquisition, statistical analysis, or interpretation of the data, review, and approved the final version of the manuscript.

## Supplementary material

Supplementary Figure 1 Preliminary diagnosis and classification of benign and malignant diseases by using the method of ROSE during EBUS-TBNA. Red, ROSE; Green, non-ROSE.

## Abbreviations

ROSE: Rapid On-site Cytologic Evaluation
EBUS-TBNA: Endobronchial Ultrasound-guided Transbronchial Needle Aspiration
CT: Computerized Tomography
PPV: Positive Predictive Value
NPV: Negative Predictive Value.
