# Supplementary figures and images for: Value of transbronchial needle aspiration combined with a rapid on-site evaluation of cytology in the diagnosis of pulmonary lesions
Source: Front Med (Lausanne). 2022 Oct 5;9:922239. doi: 10.3389/fmed.2022.922239 (PMC9579285; doi:10.3389/fmed.2022.922239)

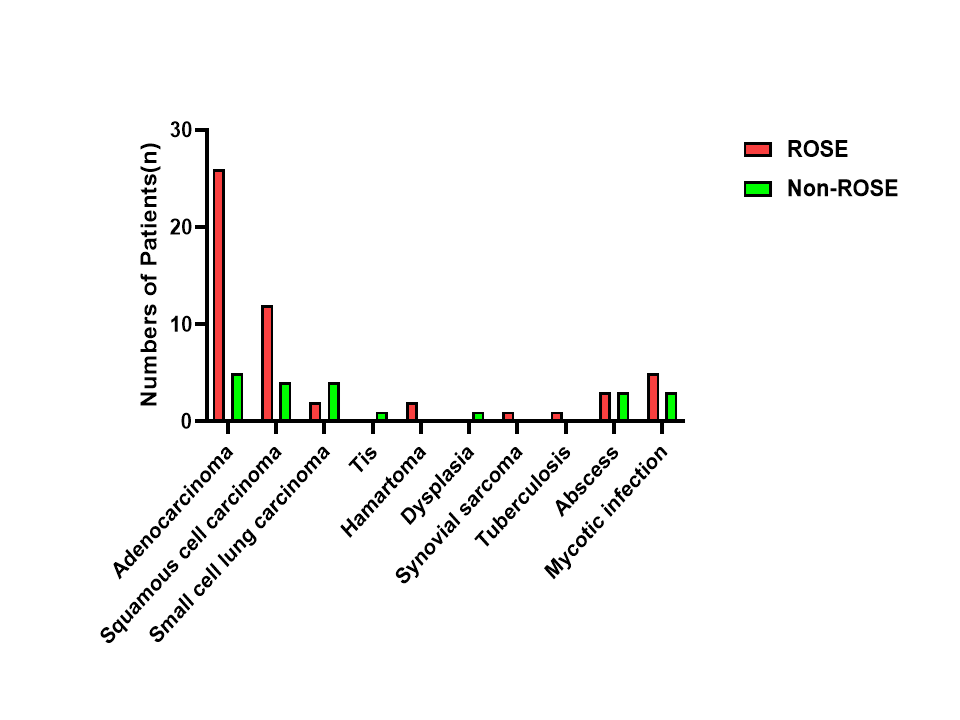

Supplement: Supplementary Figure 1 — Preliminary diagnosis and classification of benign and malignant diseases by using the method of ROSE during EBUS-TBNA. Red, ROSE; Green, non-ROSE. [file Image_1.TIF]
